# The first reported values of microplastics in prostate

**DOI:** 10.1186/s12894-024-01495-8

**Published:** 2024-05-14

**Authors:** Erhan Demirelli, Yalçın Tepe, Ural Oğuz, Handan Aydın, Murat Kodat, Doğan Sabri Tok, Mehmet Giray Sönmez, Ercan Öğreden

**Affiliations:** 1 Department of Urology, Giresun University Faculty of Medicine, Giresun, Turkey; 2 Department of Biology, Giresun University Faculty of Arts and Scienc, Giresun, Turkey; 3 Department of Urology, Necmettin Erbakan University Meram Faculty of Medicine, Konya, Turkey

**Keywords:** Prostate, Microplastic, Polyamide, ATR-FTIR spectrophotometry

## Abstract

**Background:**

Microplastics are ubiquitous, widespread environmental pollutants with unavoidable human exposure. Herein, it was aimed to investigate the presence of microplastics in prostate tissue.

**Methods:**

Prostate tissues from 12 patients who underwent Trans Urethral Resection of the Prostate (TUR-P) were analyzed to investigate the presence of microplastics. Initially, the prostate tissues were analyzed for microplastic particles using a light microscope after extraction. Subsequently, the chemical composition of the particles found in the prostate tissues was characterized using Attenuated Total Reflection-Fourier Transform Infrared (ATR-FTIR) spectrophotometry.

**Results:**

Microplastic particles of various types were detected in 6 out of 12 patients. All detected plastic particles in this study were microplastics, with sizes below 26 μm in size. These microplastics exhibited different shapes as pellets, spheres or fibers. Overall, among the 12 analyzed prostate tissue samples, four different types of plastic were identified in six samples. The most common type of microplastic detected was Polyamide (Nylon 6), found in samples from three patients. Other detected types, Polypropylene, Polyacrylic Acid and Poly (dimethylsiloxane) were each determined in samples from one patient.

**Conclusions:**

This is the first study to demonstrate the presence of microplastics in prostate tissue, serving as an exploratory investigation, which can trigger further research to validate the results in a larger patient cohort.

## Introduction

Microplastics are ubiquitous, widespread environmental pollutants with unavoidable human exposure [1]. Plastics are categorized according to their size of 1 to 1000 nm; 1 to 1000 μm ; 1 to 10 mm; and 1 cm and larger, as nanoplastics, microplastics, mesoplastics and macroplastic, respectively [2]. The world total plastics production has increased from 1.7 million tons in 1950 to ∼ 400 million tons in 2023 [3]. Excluding almost 1% bioplastics, the most part of them are not biodegradable, therefore litter the environment, part of which accumulate in living organisms especially aquatic animals [4]. Currently, researches are rapidly carrying on the detection of microplastics in marine water [5], sediment [6], freshwater [7], sea salt [8, 9], foodstuff [10], honey [11], seafood [12, 13], fish [14], etc. all around the world. Besides the detection in the gastrointestinal tract of marine animals such as mussel [13], more importantly microplastics and nanoplastics have been found in various mouse cells and tissues [15]. Undoubtedly, microplastics currently contaminate human food and their presence in various parts of the human body is reported, however little information is provided about their public health effects [16]. Human exposure to microplastics can be caused not only by ingestion but also by inhalation and dermal route [17]. Airborne microplastics are released from wide range of sources, mainly abrasion of synthetic materials and city dust [18]. Personal care and cosmetic products, which human use in daily life such as shampoos, soap, toothpaste, eyeliners, lipsticks, deodorant etc. contain plastic particles [19]. Microplastics fibers with the size of 250 μm have been spotted in human lung biopsies even in cancer tissues [20]. Bisphenol A, a chemical use primarily in the production of polycarbonate plastics and epoxy resins, have been detected in mothers’ breast milk and infant urine samples [21]. Scientists from Austria have identified nine different microplastic types in human stool with polypropylene and polyethylene terephthalate being the most abundant [22]. Italian researchers have also demonstrated the presence of microplastics in human placenta, urine and semen [23–25]. Additionally, the presence of microplastics has been recently demonstrated in meconium [26], colectomy [27], cardiac tissues [28], saphenous vein tissue [29] and cirrhotic liver samples [30]. More recently, the presence of microplastics has been confirmed in urine and kidney tissues by a group of researchers from Italy [31]. A recent study found microplastic particles in about 80% of people tested for the presence of plastic in human blood. Human blood may transport the plastic particles to the whole body and organs [32]. As it was demonstrated in the lung tissue and kidney [20, 31], microplastics particles might also be accumulated in other solid organs such as prostate tissues because of their dynamic structures; they go through continual renovation during the course of life. Thus, based on this idea the present prospective case study was performed to detect whether there is microplastic in human prostate tissue.

## Methods

### Patients characteristics

After obtaining Institutional Ethics Committee approval of Selçuk University (Approval number: 2021/3051), a prospective data analysis was performed with 12 patients complaining of lower urinary tract symptoms. The participants in this study have given written informed consent to publication of their case details. All patients were residents of Giresun and surrounding areas, with 7 from the city center and 5 from the countryside. Detailed demographic and background data of the patients were recorded, including their dietary habits (Table 1). None of the patients followed a vegetarian diet, consumed fast food, chewed gum, or used cosmetic products. Only one patient had habit of drinking alcohol. These characteristics are specific to patients’ ages and rural lifestyles.

**Table 1:** Characteristics of the study cohort

Characteristic	Value
Participants, n	12
Age	70 ± 9.8 (57 - 88)
Body Mass Index, BMI	24 ± 3.4
Smoker	4 (2 - 20 years, 1 - 2 packet per week)
Alcohol intake	1 (20 years)
Diet	
Vegetarian diet	none
Seafood meals	12 (1 - 3 per week)
Fast food	none
Plastic-wrapped food	12
Drink in plastic bottles	12
Chewing gum	none
Teeth brushing	8 (daily)
Dental prosthesis	5
Cosmetic product use	none

### Surgical technique

Presence of plastic particles in the prostate was investigated in 12 patients underwent prostate surgery at Giresun University Giresun Training and Research Hospital. All patients underwent Trans Urethral Resection of the Prostate (TUR-P) procedure under either general or spinal anesthesia at supine position. Surgical procedures followed general rules of surgical interventions with strict attention to prevent potential microplastic contamination. Surgical staff wore sterile, non-latex surgical gloves (Encore non-latex, Ansell, Brussels, Belgium). The tissues resected were removed through the metal resectoscope and then enclosed in a metal sieve. The surgery staff wore sterile non-latex surgical gloves (Encore non-latex, Ansell, Brussels, Belgium) to handle, the tissues, ensuring sterility. The tissues were then carefully placed into the sterile glass jars without touching. To avoid microplastic contamination, only the surgeon handled the prostate tissues during the surgery. Finally, the samples were immediately transferred to Giresun University Biology Laboratory for further analysis. Prior to experimentation prostate tissues of the patients were stored at -20 ^o^C in the sterile glass jars.

### Extraction and Microscopy

The protocol of Karami et al. with some modification was adopted for extraction [33]. Prior to extraction, all solutions, including ethanol and high liquid pressure chromatography (HPLC) grade distilled water were filtered through a 2.5 μm Whatman filter paper. Glassware and metal laboratory materials were washed and rinsed twice with HPLC grade distilled water and ethanol consecutively, and then dried in oven. The extraction procedure was performed in a sterile laminar flow cabinet (SEM, Turkey) to prevent potential contamination from air-suspended microplastics. Samples were placed in individual glass flasks and labelled accordingly. For each sample, 50 ml of 10% potassium hydroxide (KOH) solution was added to the glass flasks. The samples were then incubated at 40 °C to speed up the digestion process with daily shaking. Glass flasks were immediately sealed to prevent possible fiber contamination and kept in an oven at 40 °C for 3–5 days. The suspension was sonicated at 50 Hz for 5 min and shaken at 200 rpm for 5 min. Subsequently, the suspension was centrifuged at 500xg for 5 min, and the supernatant was collected and filtered through 2.5 μm Whatman filter membrane using a vacuum pump connected to a filter funnel. The filtrate was analyzed for the presence of microplastic particles under light microscopy (Olympus CKX41) using 10× objectives, and images were photographed with a camera (Celestron) connected to the microscope. Data were expressed as “Mean ± s.e.m.”.

### ATR-FTIR spectrophotometry

The chemical composition of particles present in prostate tissues obtained from 12 patients was characterized by using ATR-FTIR spectrophotometry (Shimadzu IR Prestige-21) as it is very precise and accepted methodology in the literature of microplastics studies [34]. Three consecutive spectra were obtained from each sample. The measurement range was 4000 –400 cm^− 1^ with a resolution of 4 cm^− 1^ for each spectrum, averaging 64 scans and background scans were obtained before scanning each homogenized samples. All spectra were analyzed by the software Essential FTIR v3.50.205 (Operant LLC), which enabled data normalization and base-line correction. It is recognized that UV radiation, physical or chemical degradation, and enzymatic digestion can affect the spectra of microplastics [35]. Particular effort was made to apply similar force and contact levels on the samples to minimize potential differences in peak intensity due to changes in applied contact force in FTIR-ATR analysis [36]. The most common polymers such as polyethylene, polypropylene, polyethylene terephthalate, polystyrene, polyamide, polyvinyl alcohol, polyvinyl chloride, polyester, acrylonitrile butadiene styrene, nitrile, latex, polymethyl methacrylate, polyurethane, polyoxymethylene and polycarbonate were investigated. Spectra of distilled water, saline and 5% dextrose were also obtained to avoid the risk of plastics contamination from infusion or injection solutions for drug administration during surgery. In ATR-FTIR spectrophotometry, Polyamide was shown to give absorption bands at 1462, 1438 and 1371 cm^− 1^ corresponding to C-CH stretching and 1417 cm^− 1^ CH_2_ bend, while Polypropylene was shown to give absorption bands at 1455, 2838 and 2917 cm^− 1^ attributed to C-H stretching [35, 37–41]. Spectra with a quality index more than 75% were accepted for microplastic presence and were cross-checked with spectra available in the literature.

### Quality control

During surgery several medications diluted in distilled water, saline or 5% dextrose were administered to the patients via intravenous infusion or bolus injection.

To ensure that microplastics from infusion or injection sets did not contaminate prostate tissues, all these solutions underwent ATR-FTIR spectrophotometry for quality assurance check.

Solutions without samples, run through the measurements to ensure sterile conditions and absence of plastic contamination. No plastic derivatives were detected in the spectra of distilled water, saline, and 5% dextrose, which were used to control possible plastic contamination from infusion or injection solutions for drug administration during surgery. During the laboratory studies, protective equipment, including gloves and full-body aprons, was worn to prevent potential plastic contamination from clothing and air. Throughout the entire study, stringent quality control measures were implemented to prevent cross contamination in all analyses processes, including during prostate surgeries, transportation of prostate tissues to the laboratory, tissue preservation until extraction, addition of extraction solution, dissolution of organic fractions, filtration and identification of microplastics both under microscope and with FTIR spectrophotometer. Filtered filter papers are stored in autoclaved glass petri dishes until further analysis. Blank experiments were performed in triplicate using extraction solutions placed in empty tubes to identify possible cross-contamination of airborne microplastics during each analysis in the laboratory. These blank tubes were analyzed in parallel with the samples and no microplastics were detected in the blanks.

## Results

**Fig. 1:**
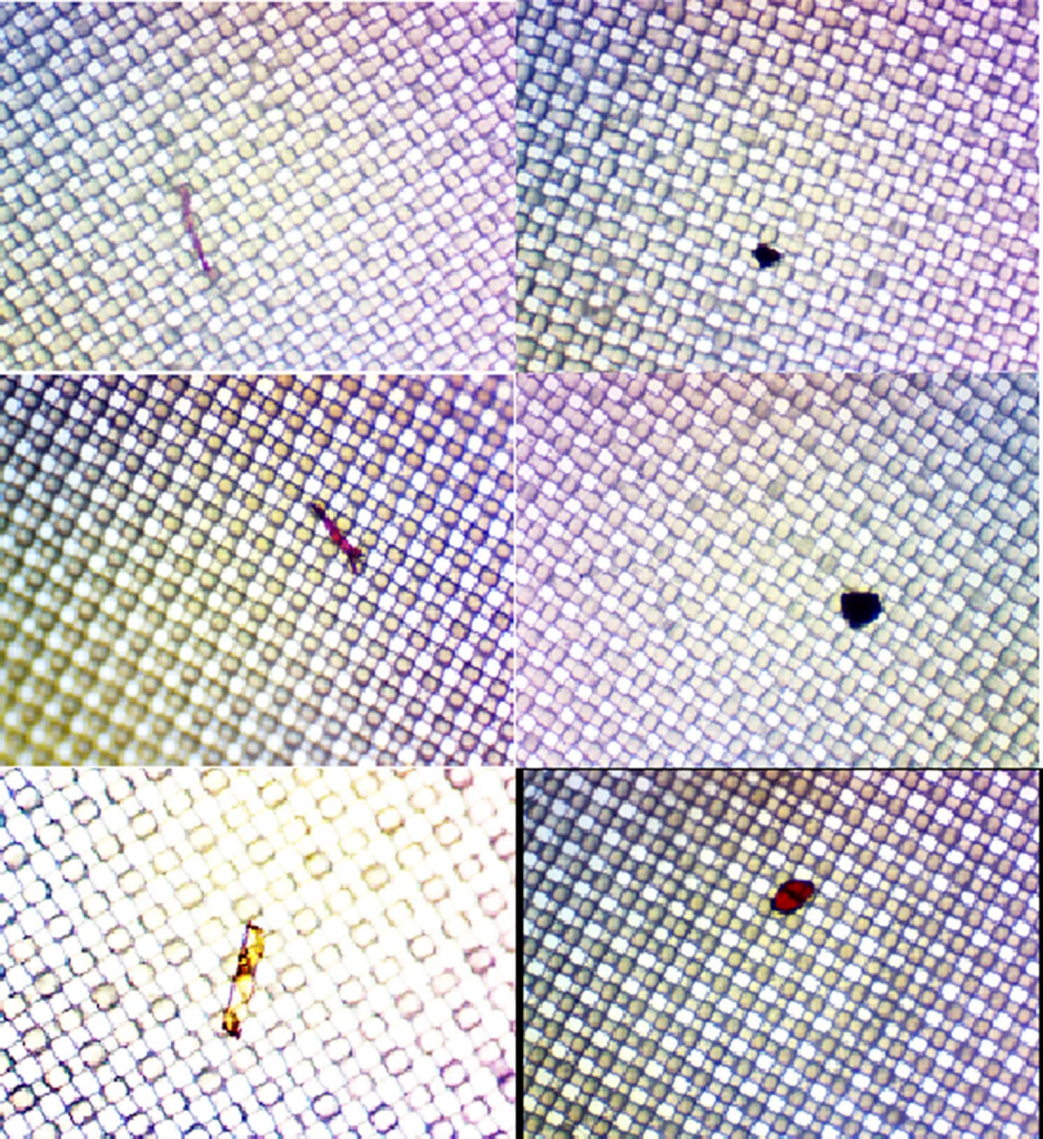
Microplastics particles in prostate tissue samples

The age and body mass index (BMI) of the patients ranged from 57 to 88 years (mean 70 ± 9.8, *n* = 12) and 21.22 to 32.17 (mean 24 ± 3.4, *n* = 12), respectively. Microplastic particles of various types were detected in samples from 6 out of 12 patients and the rest of them were free of microplastics. The average of 21.5 ± 10.13 microplastic particles ranging 2.5 μm to 26 μm in size were counted per prostate tissue samples, with a mean volume of 70 ± 19.9 cc. Because the tissues extracted during surgery were examined for pathologic evaluation, the least amount of tissue possible was used for samples. Mean prostate amount removed during the surgery was 21.09 ± 7.67 (12.6–41) gr and mean tissue amount used for samples was 1.73 (0.58-5,27) gr. The identified microplastics were generally shaped as pellets and rarely as spheres or fibers as shown in the Fig. 1.

Prostate samples harvested from patients residing in urban areas exhibited higher presence of microplastic particles compared to samples from patients living in rural areas.

**Fig. 2:**
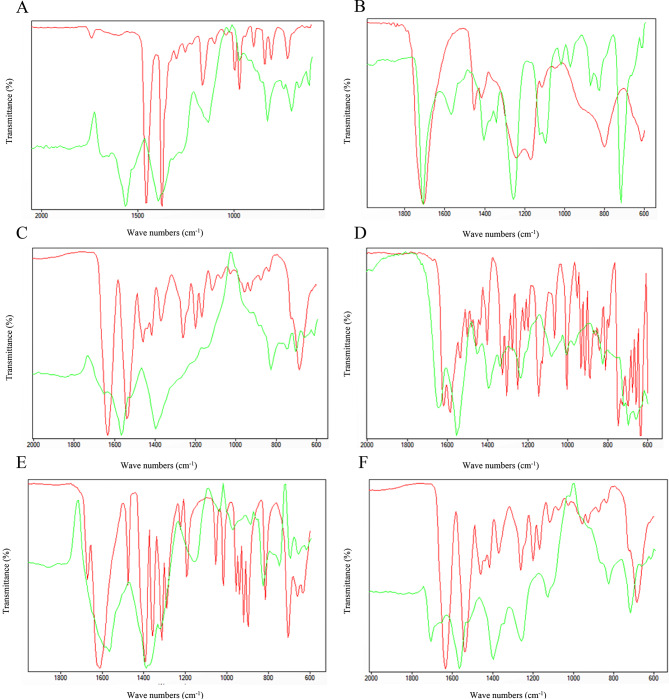
ATR-FTIR spectra showing relative frequency of microplastics, (Red: Reference spectrum, Green: Spectra from samples). (**A**). Polypropylene obtained from 10th patient. (**B**). Polyacrylic Acid obtained from 11th patient C. Polyamide (Nylon 6) obtained from 12th patient. (**D**). Poly(dimethylsiloxane) obtained from 6th patient. (**E**). Polyamide (Nylon 6) obtained from 4th patient. F. Polyamide (Nylon 6) obtained from 9th patient

Prostate tissue samples were measured in ATR-FTIR spectrophotometry in order to identify their chemical compositions. Four different microplastic derivatives were identified in 6 patients. Polyamide (Nylon 6) was the most common type of microplastics detected, found in samples from three patients. Other detected types included Polypropylene, Polyacrylic Acid and Polydimethylsiloxane, each identified in samples from one patient (Fig. 2).

Human exposure to microplastics through their diet is evident and seafood with the high ratio of microplastic particles creates a major risk in terms of food safety [42]. All of our patients consume seafood meals one to three times a week because this habit exists in the traditions of the coastal people. On the other hand, none of them eat fast food or use cosmetic products may be related to their old age and peasant lifestyle (Table 1). Moreover, five of the patients were detected with dental prosthesis and all patients declared that they consumed plastic-packaged food and drank from plastic bottles which could be considered as a risk factor for systemic plastic exposure.

## Discussion

Microplastics are ubiquitous on land, in freshwaters, and oceans, with their distribution spanning the globe [43]. Researchers frequently investigate the presence of microplastics in various environments such as oceans, sediments, soils, seafood, etc. In recent years, their accumulation in the human body has aroused worldwide curiosity and various studies have emerged on this subject. This study was the first to report the accumulation of microplastics in prostate tissue, aiming to determine plastics particles smaller than 26 μm both chemically and physically.

The presence of microplastics in the investigated patients appears to be convincingly linked to numerous sources of anthropogenic pollution such as contaminated water and food, dental prosthesis, etc. In the present study, 5 of the patients had dental prosthesis. Cox et al. have recently estimated that human consumption of microplastics ranges from 39,000 to 52,000 particles annually with an additional 90,000 microplastics in the case of consuming bottled water [44]. In this study, all patients declared that they consumed plastic-packaged food and drank from plastic bottles. However, only one sample displayed polypropylene and none of our prostate samples showed polyethylene terephthalate, suggesting it may not accumulate in prostate tissues.

Microplastics can enter the body through oral ingestion, inhalation or transdermal routes, with oral ingestion considered the most common route of exposure. Microplastics contaminated in food can be absorbed and circulate through the body. It has been reported that microplastic particles smaller than 150 μm in diameter can be absorbed from the human intestines [44]. However, there is limited data available regarding transdermal or inhalation absorption. Schwabl et al. have shown a median of 20 microplastic particles ranging from 50 to 500 μm in size in human stool [22]. The relatively higher number of particles observed in the present study compared to the stool study, might be related to the long-term accumulation of microplastics in prostate tissues. Moreover, particles smaller than 50 μm in size were not investigated in stool samples. It has been shown that Polystyrene particles ranging from 1 to 10 μm in diameter can be absorbed from the intestines of mice after oral administration [41]. In addition, evidence supports the presence of microplastics in the air, suggesting exposure to microplastics via inhalation in human [45]. Plastic microfibers with 135 μm in length have been observed in lung tissues obtained from patients with lung cancer [20]. In the present study, microplastic particles smaller than 26 μm in size were observed, consistent with findings in the literature. Due to limitations in the extraction of microplastics from prostate tissues as mentioned in methods section, we were unable to detect particles smaller than 2.5 μm in size. This raises questions about the potential presence of smaller particles.

The prostate is located inferior to the bladder and covers the proximal urethra within the true pelvis. It is composed of two cellular compartments including stromal and epithelial cells. A fibrous capsule wraps the prostate gland, with the nerves and prostatic vascular plexus. The prostate is not only important for the male reproductive system, but also important for lower urinary system symptoms in advanced age and important that it is the origin of the most common male urinary system cancer [46, 47]. There are several articles that have demonstrated the presence of microplastics in various parts of the human body. The presence of microplastics has also been shown in semen, some of which components are produced by the prostate [24]. However, the presence of microplastic in the prostate has not been investigated before. The presence of microplastic in the prostate tissue was revealed for the first time in the present study. The relationship between the microplastic and prostatic diseases is also needs to be investigated in future studies.

This study was conducted with a limited number of researchers to minimize potential plastic contamination during the analyses. However, there are several limitations to consider. First, the study population was small. Second, the patients that participated were relatively older individuals; thus, the presence of microplastic particles in the younger and healthier individuals remained unknown. Third, all patients were residents of Giresun and surrounding areas, as our hospital serves mostly for the cities of East Black Sea Region. Thus, our results may be representative of only a limited region. Forth, the extraction method used needs some improvements. This study can be considered as an exploratory study, and may trigger further research to validate the results of this study in a larger cohort of patients. These findings suggest that many efforts should be made to reduce human exposures to plastic derivatives. The identification of microplastics into prostate tissues opens new perspectives on human exposure to plastics. In addition, from a clinical point of view, the detection of microplastics contamination in the prostate or other tissues (i.e., soft tissues, organs, and blood) could become a powerful tool for assessing health status. Further in vivo and in vitro studies are necessary to elucidate the molecular mechanism by which microplastics accumulate in prostate tissues in humans.

## Conclusions

In the present study, the presence of microplastics in prostate tissue was demonstrated for the first time. The identified microplastics generally appeared as pellets, with spheres or fibers observed less frequently. Overall, four different types of plastics were identified in half of the 12 patients studied. Polyamide (Nylon 6) was the most common type of microplastics detected, while other types included Polypropylene, Polyacrylic Acid and Polydimethylsiloxane. The presence of microplastics in many environments and even in different organs and systems of the human body is quite worrying. The gaps and public health threats behind this novel topic need to be addressed and investigated in the future.

## Data Availability

The datasets used and/or analysed during the current study are available from the authors on reasonable request.
